# The crystal structure of fibroblast growth factor 18 (FGF18)

**DOI:** 10.1007/s13238-014-0033-4

**Published:** 2014-03-26

**Authors:** Alan Brown, Lucy E. Adam, Tom L. Blundell

**Affiliations:** Department of Biochemistry, University of Cambridge, Cambridge, CB2 1GA UK

**Dear Editor**,

Fibroblast growth factors (FGFs) regulate a plethora of critical processes in development (Beenken and Mohammadi, 2009). These processes are mediated by signaling through four FGF receptors (FGFR1–4), which are high-affinity cell surface receptor tyrosine kinases. Receptors 1–3 undergo alternative splicing to generate b- and c-isoforms with altered ligand specificities and affinities. Ligand to receptor binding is not unique and one receptor can be activated by several FGFs. Heparan sulfate (HS) proteoglycan, a variably polysulfated glycosaminoglycan related to heparin, is an essential requirement for FGF signaling with FGFs varying in their specificities for different HS sulfation patterns (Gallagher et al., 1992).

The 18 mammalian FGFs that are capable of signaling through FGFRs share a conserved β-trefoil fold and are grouped into 6 subfamilies (Itoh and Ornitz, 2004). FGF18 belongs to the paracrine-acting FGF8 subfamily, which contains three members in humans: FGF8, FGF17, and FGF18. The biological activities of FGF8 and FGF17 are regulated by alternative splicing with four isoforms of FGF8 (a, b, e, and f) and two of FGF17. In contrast, FGF18 does not undergo alternative splicing.

FGF18 has a number of functions in the developing and adult organism including a key role in skeletal development (Haque et al., 2007) and has undergone clinical trials for the treatment of osteoarthritis (Merck KGaA). Pathologically, FGF18 is implicated in colorectal (Shimokawa et al., 2003) and ovarian cancer (Wei et al., 2013) and has been proposed as an early marker and potential anticancer drug target.

*In vivo*, FGF18 is expressed as a precursor polypeptide of 207 residues with the initial 27 hydrophobic residues forming a cleavable signal peptide. Residues 50–194 were expressed in *Escherichia coli* and purified to homogeneity. The interaction between FGF18 and heparin oligosaccharides was examined using isothermal titration calorimetry (ITC), Fig. S1A. FGF18 binds heparin oligosaccharides of 6 dp with an affinity of 2.2 ± 29.2 μmol/L. The interaction is enthalpy-driven (ΔH = −8.6 kcal·mol^−1^) with a negative entropic contribution (TΔS = −1.0 kcal·mol^−1^) giving an overall free energy (ΔG) of −7.6 kcal·mol^−1^. A heparin oligosaccharide of 8 dp was sufficient to dimerize FGF18 (Fig. S1B), as observed for FGF1 (Brown et al., 2013).

FGF18 was incubated stoichiometrically with heparin (6 dp) and the complex isolated prior to crystallization in a mother liquor of 0.1 mol/L MES pH 6.5, 0.2 mol/L ammonium sulphate and 26% PEG 5000. FGF18 crystallized in the primitive monoclinic spacegroup P2_1_ with four FGF18 molecules in the asymmetric unit. Initial phases were provided using molecular replacement with the FGF8b structure (PDB ID: 2FDB) and refined to 2.7 Å. Data collection and refinement statistics are shown in Table S1, with an example of the electron density in Fig. S2.

The final model consists of residues 50–179 and has a β-trefoil core formed from ten anti-parallel β-strands arranged in three groups of three or four strands connected by tight turns and loops (Fig. 1A). Two strands from each group come together to form a β-sheet barrel of six antiparallel β-strands. There is a disulfide bridge between Cys109 and Cys127 that joins the β6 strand with the β7–β8 loop, helping to stabilize it. These cysteines are conserved throughout the FGF8 subfamily with Cys127 conserved throughout the entire FGF family. This disulfide bond restricts FGF18 to exerting an extracellular function only, unlike other FGF members that have intracellular roles. The four FGF18 molecules in the asymmetric unit have root mean-square deviation (RMSD) of 0.30 Å^2^ (Cα atoms). Differences are mainly at the crystal contact sites with greatest variation in the β4–β5 loop, residues 90–96 (Fig. 1B), which in the FGF8 subfamily contains an extra serine residue that presumably increases its flexibility.

**Figure 1:**
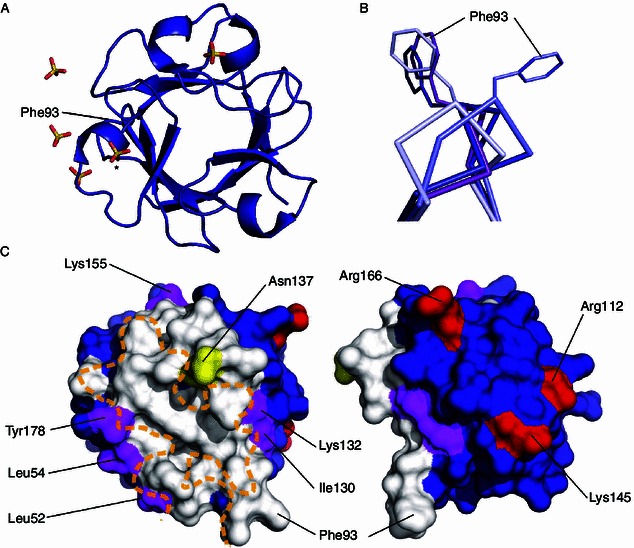
**The structure of FGF18**. (A) Cartoon representation of the FGF18 crystal structure of protomer A. Sulfate ions are shown in stick representation with sulfur in yellow and oxygen in red. The position of a sulfate found in another protomer in the asymmetric unit is demarcated with an asterisk. (B) Structural heterogeneity in the β4–β5 loop from the four molecules in the asymmetric unit with protomer A shown in dark blue. (C) Two views of the surface of FGF18. Residues equivalent to FGFR2c-binding residues in FGF8b are shown in white with interface residues that differ in magenta. Asn139, a putative glycosylation site in the center of the FGFR interface, is shown in yellow. A dashed orange line outlines the positions of ‘hot spot’ residues at the FGF8b-FGFR2c interface. FGF18 polymorphisms that occur on the surface of the structure are shown in red. The position of Phe93 is indicated in each panel

The members of the FGF8 subfamily differ in their specificities and affinities for FGFRs, although all lack affinity for FGFR b-isoforms. In comparative SPR assays, FGF18 has been shown to have highest affinity to either FGFR3c (Hoshikawa et al., 2002) or FGFR1c (Olsen et al., 2006). The latter study shows FGF18 has weaker affinity for each receptor than FGF8b. By comparing the structure of FGF18 with the binary complex of FGF8b-FGFR2c insights into their different affinities are possible. The FGF18-FGFR2c complex was modelled by superposition of FGF18 onto FGF8b (RMSD = 0.95 Å^2^) in the FGF8b-FGFR2c binary complex (Olsen et al., 2006), Fig. S4A. The model demonstrates how the extended and hydrophobic β4–β5 loop of FGF18 is important for conferring FGFR binding specificity as it engages the hydrophobic groove in the D3 domain of FGFR c-isoforms. In the FGFR b-isoform this region predominantly contains hydrophilic residues that would repel FGF18 (Fig. S4C and S4D).

Of the interface residues for which structural information is present for both FGF8b and FGF18 only six vary, Figs. 1C and S3. All of these residues are outside predicted ‘hot spots’ that are residues that make a dominant contribution to the free energy of binding, typically ΔΔG ≥ 2 kcal·mol^−1^, and that if mutated can have a substantial effect on the affinity of the interaction. Many of these mutations, for example Arg155 to Lys155, preserve physicochemical properties. Cumulatively, these changes may explain the different specificities/affinities of FGF18 and FGF8b, however, it is likely that differences in the N- and C-termini that are not resolved in the FGF18 crystal structure, contribute substantially. The FGF18 structure is missing the initial 23 residues of the mature polypeptide and 28 residues from the C-terminus. In the crystal structure of FGF8b-FGFR2c, the FGF8b N-terminus is ordered and tethered to the β4 strand and to the β4–β5 loop within the β-trefoil core via numerous hydrophobic contacts and hydrogen bonds. Residues, especially Phe32 and Val36, from this structured N-terminus contact the hydrophobic groove in the D3 domain of the receptor and help confer specificity towards the c-isoforms. Phe32 has been shown to be key in conferring the differing affinities of FGF8a and FGF8b for the FGFR (Olsen et al., 2006). There are 3 residues in this N-terminal section that differ between FGF18 and FGF8b (Arg34 [Thr in FGF8b], Val37 [Leu] and Lys49 [Arg]), Fig. S4B. The change from threonine in FGF8b to arginine in FGF18, could reduce hydrophobic packing with the hydrophobic groove of the D3 domain, and therefore explain the weaker affinity FGF18 has for each of the c-isoforms compared to FGF8b.

HS is a necessary component of paracrine FGF signaling complexes. The interaction with FGFs is dependent on formation of suitable conformational and charge characteristics that are complementary to the binding sites on the proteins formed. However, these requirements can be satisfied by multiple polysaccharide sequences (Beenken and Mohammadi, 2009; Rudd et al., 2010). FGF18 has a lower stringency for HS sulfation than other FGFs as it recognizes both 2-*O*-sulfated and 6-*O*-sulfated HS oligosaccharides, with a preference for 2-*O*-sulfation (Ashikari-Hada et al., 2004).

Although electron density for heparin hexasaccharide is not present, 13 sulfate ions are discernable in the asymmetric unit, corresponding to 5 unique sulfate-binding locations (Fig. 1A). The position of these sulfates, and the surface charge distribution, offers clues to the mode of HS recognition by FGF18. The position of sulfates within the FGF1 crystal structure were first used to correctly identify HS-binding residues (Blaber et al., 1996). However, it should be noted that only highly co-ordinated sulfate ions are likely to be ordered in the crystal structure, and in the context of heparin, further sulfate-binding positions are possible. A recent study has used a ‘protect and label’ method to identify lysine residues that participate in heparin-binding in FGF18 (Xu et al., 2012). From this, they identified both a canonical and a secondary heparin-binding site in FGF18. In our structure, sulfate ions are bound to the canonical binding site, but not to the proposed secondary site. A detailed view of the atoms involved in contacting the sulfates is shown in Fig. 2 along with a comparison of the positions of sulfates from heparin in FGF1 and FGF2. Three of the five sulfate ions are clustered in a location distinct from the heparin-binding sites of FGF1/2 (Fig. 2D–E), but occupy a continuous stretch of basic residues that is, in part, formed by the lysines identified as interacting with heparin by Xu et al. (2012), and could represent an extension of the canonical site. Whether these sites would be occupied in solution by a longer stretch of HS or whether two HS molecules could bind simultaneously remains to be fully determined. However, the ITC data (Fig. S1) suggests that FGF18 cannot bind two molecules of heparin oligosaccharides of 6 dp or 8 dp simultaneously.

**Figure 2:**
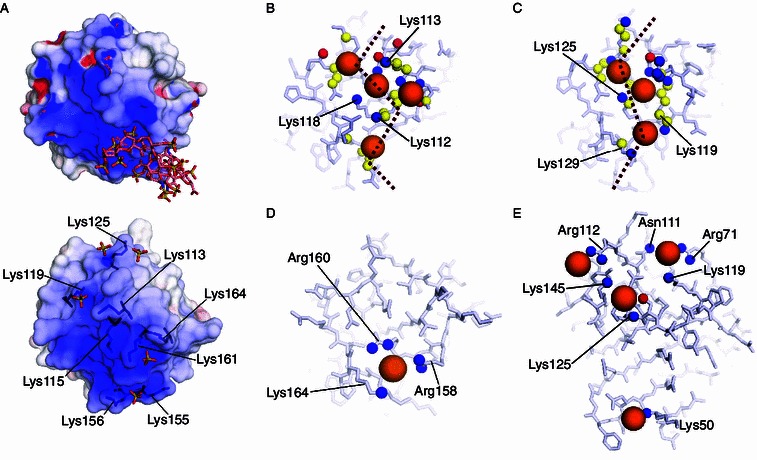
**Insights into the FGF18-HS interface from bound sulfate ions**. (A) Surface electrostatic potentials were calculated for FGF1 (top) and FGF18 (bottom). Red represents −5 e per Å and blue represents +5 e per Å. Heparin bound to FGF1 and sulfate ions bound to FGF18 are shown in stick representation. Lysine residues from FGF18 that contact heparin in solution (Xu et al., 2012) are shown; Lys155, 156, 161, and 164 constitute the canonical HS-binding site with an equivalent in FGF1, with Lys113, 115, 119, and 125 unique to FGF18. The heparin-binding interfaces of FGF1 (B), and FGF2 (C), compared to the location of sulfates bound to FGF18 (D and E). Sulfate ions or sulfates from heparin oligosaccharides are shown as large orange spheres. Interacting atoms with an inter-atomic distance equal to or less than 3.6 Å are displayed as small spheres. Carbon atoms are colored yellow, oxygen atoms are colored red, and nitrogen atoms colored blue. A dashed purple line shows the trajectory of the heparin sugar backbone. Key interacting residues are labelled

Alongside the lysine residues identified by Xu et al. (2012), a number of arginine residues are involved in binding the sulfate ions, more than identified in the FGF1/2-heparin interfaces. The enthalpy of arginine residues for heparin anions is 2.5 times greater than for lysine residues, due to the guanidine groups producing stronger hydrogen bonding and a more exothermic electrostatic interaction (Fromm et al., 1995). It is possible that the higher affinity provided by arginine residues in a HS-binding interface could explain why FGF18 has a lower stringency for HS sulfation than other FGFs.

When secreted from mammalian cells, FGF18 acquires 4 kDa of N-linked oligosaccharide (Hu et al., 1998). FGF8b subfamily members have an invariant potential glycosylation site at Asn137, with FGF18 having a second site at Asn39. In the FGF18 crystal structure, Asn137 is surface exposed and would interact with Arg251 and Pro170 in FGFR2c if FGF18 is modelled according to the known FGF8b-FGFR2c complex (Fig. 1C and S4A). Asn39 is not present in the structure, but from the sequence-to-structure alignment (Fig. S4B) would correspond to FGF8b Glu38. This residue faces away from the FGFR2c interface and is unlikely to influence binding. While it is possible that glycosylation at Asn137 could influence the ability of the FGF8 subfamily to bind FGFRs, it has been reported that glycosylation is not critical for FGF18 mitogenic activity on fibroblast cell lines (Hu et al., 1998). Other FGFs are glycosylated although its importance to function remains to be fully elucidated.

A number of polymorphisms have been identified in FGF18, including mutations in cancer patients (Table S2). These polymorphisms were mapped to the structure of FGF18 to analyze possible effects on protein interaction interfaces or protein stability (Fig. 1C). Leu16Met is in the N-terminal signal peptide and therefore does not have any effect on the mature polypeptide. Arg49His is not in the FGF18 crystal structure, but is structurally equivalent to Arg49 in the FGF8b structure, which while adjacent to two interacting residues the sidechain of Arg49 does not contribute to the interface, and as such a mutation to histidine is unlikely to alter the binding to FGFR. Arg112 and Lys145 may be located in the HS-binding interface based on their interaction with sulfate ions and their mutation to histidine or glutamine could have an effect of lowering affinity for HS. Arg166, although solvent-exposed, is outside the putative HS- and FGFR-binding interfaces and a polymorphism here is unlikely to have a large effect on FGF signaling. Phe153 is solvent inaccessible and contributes to the hydrophobic core of the protein. Leucine has a similar hydrophobicity and therefore the mutation is unlikely to considerably destabilize the protein.

In summary, we have solved the structure of the clinically important FGF18 protein. The position of sulfate ions bound to FGF18 provides insight into the putative HS-binding site and allows comparison with the prototypical FGFs, FGF1, and FGF2. The structure also reveals the molecular mechanism behind the specificity of FGF18 for FGFR c-isoforms and the reduced affinity for these compared to FGF8b. We propose that based on the location of the Asn137 glycosylation site within the FGFR-binding interface, glycosylation of FGF8 subfamily members could play a role in modulating affinity towards FGFRs.

## Electronic supplementary material

Below is the link to the electronic supplementary material.

Supplementary material 1 (PDF 19533 kb)
