# Supplementary material for: The crystal structure of fibroblast growth factor 18 (FGF18)
Source: Protein Cell. 2014 Mar 26;5(5):343–7. doi: 10.1007/s13238-014-0033-4 (PMC3996155; doi:10.1007/s13238-014-0033-4)
Supplement: Supplementary file 1 — Supplementary material 1 (PDF 19533 kb) [file 13238_2014_33_MOESM1_ESM.pdf]

The crystal structure of Fibroblast Growth Factor 18 (FGF18)

Supplementary information

Materials and Methods

Heparin oligosaccharides of defined length were prepared from partial heparinase digests of heparin by size-exclusion chromatography (Ostrovsky et al., 2002).

Expression and Purification of FGF18

A human gene fragment encoding FGF18 (Uniprot ID: O76093, residues 50-194) was cloned into the pBAT4 vector (Peränen et al., 1996; Ostrovsky et al., 2002) using forward primer 5' ggccatggcacagctgcgtctgtatcag 3' and reverse primer 5' ggctcgagcaccgtcgtgtatttgaagg 3'. FGF18 was expressed in *E. coli* BL21 Rosetta-gami2 (DE3) cells grown in 2xYT with 100 μg ml^{-1} ampicillin by induction with 1.0 mM IPTG overnight at 18°C. Cells were pelleted and resuspended in solubilization buffer (50 mM Tris pH 8.0, 500 mM NaCl, 1 mM EDTA, 1.0 % (v/v) Triton-X100, 10 mM β-mercaptoethanol, protease inhibitor tablet (Roche)) and lysed by sonication. The soluble fraction was obtained by centrifugation at 15,000 x g at 4 °C for 30 min and applied to a 5 ml Heparin HP column (GE Healthcare). The protein was eluted using a NaCl gradient increased to 2.0 M. The protein was desalted and buffer exchanged into 20 mM Tris pH 8.0, 150 mM NaCl. FGF18 was further purified by size-exclusion chromatography using a Superdex 75 16/60 column (GE Healthcare) equilibrated with 20 mM Tris pH 8.0, 150 mM NaCl.

Isothermal Titration Calorimetry (ITC)

ITC was performed using a MicroCal VP-ITC machine (GE Healthcare). Titrations involved the addition of 10 μl aliquots of heparin oligosaccharides via a rotating stirrer-syringe to the calorimetric cell containing 1.4 ml of FGF18 at 4 min intervals for a total of 20 injections. A constant temperature of 25°C and stirring speed of 300 rpm was maintained throughout. Heats of dilution determined in the absence of FGF18 were subtracted from the titration data before curve fitting. Additionally a 4 μl injection was discarded from each dataset to remove the effect of titrant diffusion across the syringe tip during the equilibration process. Data were modelled by non-linear least squares fitting and a full set of thermodynamic parameters derived using $\Delta G = \Delta H - T\Delta S = -RT\ln(1/K_D)$, where ΔG, ΔH and ΔS are the Gibbs free energy, enthalpy and entropy of binding respectively. T is the absolute temperature, R = 1.98 cal mol^{-1} K^{-1} is the ideal gas law constant and K_D is the dissociation constant.

Crystallization of FGF18

FGF18 was mixed in a 1:1 stoichiometry with heparin hexasaccharide and the complex purified using size-exclusion chromatography on a Superdex 200 10/30 column (GE Healthcare) equilibrated with 20 mM Tris pH 8.0, 150 mM NaCl. FGF18-hexasaccharide was concentrated to 5 mg ml^{-1} prior to crystallization. Optimal crystals were obtained in 0.1 M MES pH 6.5, 0.2 M $(NH_4)_2SO_4$ and 26% PEG 5000. The crystals were cryoprotected by the addition of 26 % (v/v) ethylene glycol (soaked for ~ 1 min) and vitrified by plunging in liquid nitrogen.

Structure determination and refinement

X-ray diffraction data were collected on a X8 PROTEUM (Bruker AXS) at a wavelength 1.54 Å. Data were indexed and scaled using PROTEUM 2 software (Bruker AXS) and structure factor amplitudes calculated from the merged intensities. To provide phases, a molecular replacement search was performed using PHASER (McCoy et al., 2007) with the known crystal structure of FGF8b as a search model (chain M from PDB ID: 2FDB). Maximum likelihood-based restrained refinement was carried out using REFMAC (Murshudov et al., 2011) with manual rebuilding in Coot (Emsley and Cowtan, 2004). The refinement strategy was optimized using PDB_REDO (Joosten et al., 2011). The stereochemistry of the structure was assessed and validated with MolProbity (Chen et al., 2009). The final refinement statistics are shown in Table 1. The structure has been deposited in the PDB with PDB ID: 4JCM).

Structure comparison

The structures of other FGFs were obtained from the Protein Data Bank (Rose et al., 2011). FGF8b-FGFR2c interfaces were analyzed using PISA (Krissinel and Henrick, 2007) with interface 'hot spot' residues identified using HSPred (Lise et al., 2011). Structural superpositions were performed using SuperPose (Maiti et al., 2004). The PDB2PQR server and the Adaptive Poisson-Boltzmann Solver (Unni et al., 2011) were used to calculate electrostatic potential. All images were prepared using PyMOL (DeLano, 2002) with secondary structure assigned with STRIDE (Heinig and Frishman, 2004).

Table 1: Data collection statistics

Data collection	
Space group	$P2_1$
Unit cell parameters (Å)	65.76 49.49 100.85<br>90 101.74 90
Wavelength (λ)	1.54
Resolution (Å)	98.74 – 2.70 (2.80-2.70)
I/σI	15.84 (2.83)
Completeness (%)	100 (100)
Data redundancy	19.30 (12.99)
R_{int}	0.1916 (0.6345)
Unique reflections	17,815 (1,819)
Mosaicity	0.41
Unit cell volume ($Å^3$)	321,396
Refinement	
$^{*}R_{cryst}/^{\dagger}R_{free}$	0.21/0.25
Number of non-hydrogen atoms	4,261
macromolecules	4,188
ligands	65
water	8
Protein residues	514
RMSD bond length (Å)	0.009
bond angle (°)	1.33
Average B factor ($Å^2$)	34.36
Ramachandran favoured (%)	99.2
Ramachandran outliers (%)	0
MolProbity Clashscore	1.76 (100th percentile)

$^{*}R_{cryst} = \sum_{hkl}||F_{obs}- |F_{calc}||/\sum_{hkl}|F_{obs}|$

$^{\dagger}R_{free}$ was calculated from 5% of the data

ID	Mutation	Sample
rs34986991	Lys16Met	
rs138337537	Arg49His	
rs201988531	Arg112His	
rs11553493	Lys145Gln	
rs141572757	Lys180Arg	
rs150911562	Pro183Leu	
232337	Phe153Leu	skin malignant melanoma
244511	Arg166Trp	prostate carcinoma

Table S2: Polymorphisms and mutations identified in FGF18. Missense single nucleotide polymorphisms were extracted from NCBI dbSNP (Sherry et al., 2001) and mutations identified in cancer samples from COSMIC (Forbes et al., 2011).

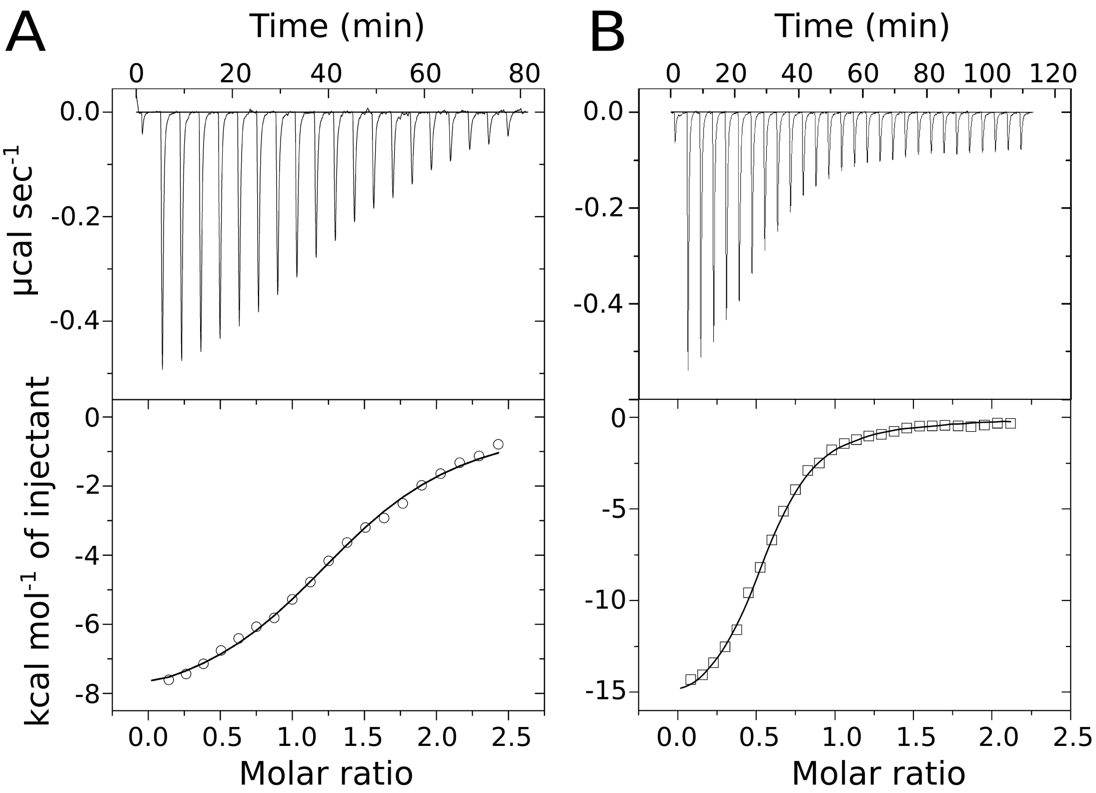

Figure S1: Analysis of FGF18-heparin interactions by ITC. Isotherms generated when 200 μM heparin oligosaccharides were titrated into 12.5 μM FGF18. (A) FGF18 forms a 1:1 interaction with heparin oligosaccharides of 6 dp. (B) FGF18 dimerizes in the presence of heparin oligosaccharides of 8 dp.

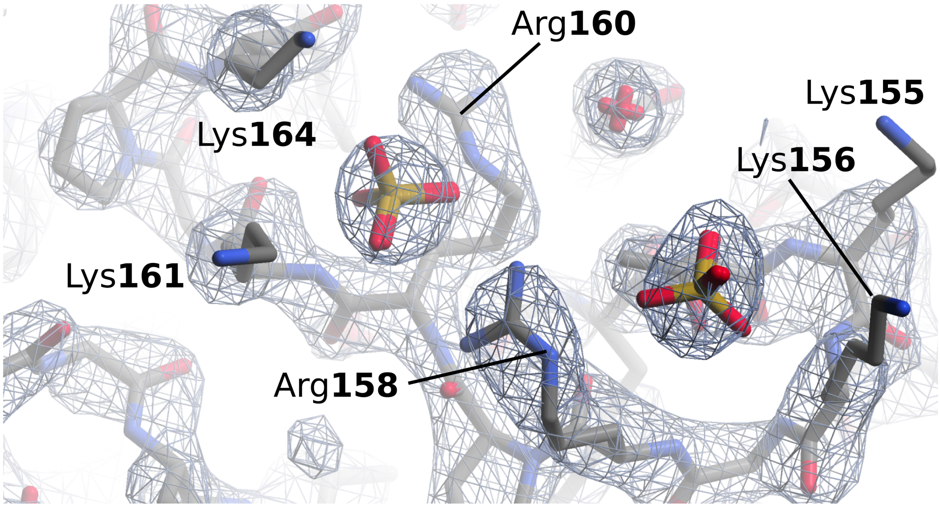

Fig. S2: Representative electron density ($2F_{obs}$-F_{calc}). Two sulfate ions bound in a highly basic pocket.

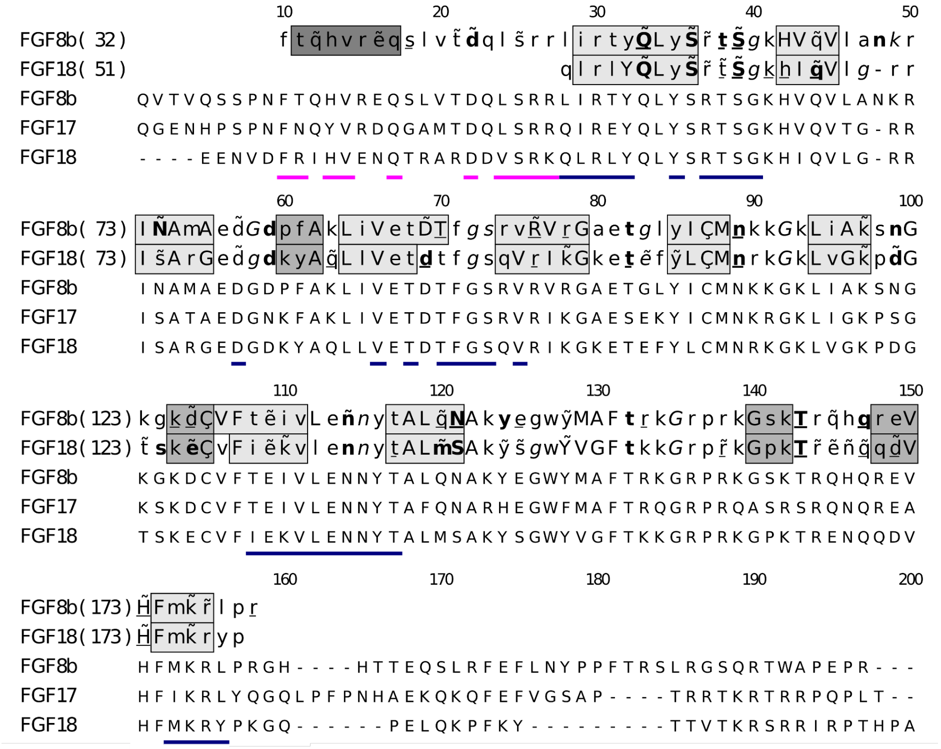

Figure S3: Sequence-to-structure alignment for the FGF8 subfamily. Full-length amino acid sequences of the mature polypeptides of the FGF8 subfamily were aligned to the structural alignment of FGF8b and FGF18. The 'canonical' isoform of both FGF8 and FGF17 from the UNIPROT database are shown. Multiple sequence and structure alignment was performed using PROMALS3D (Pei et al., 2008) and JOY was used to annotate the 3D structural information (Mizuguchi et al., 1998). Solvent inaccessible residues are shown in uppercase, solvent accessible in lower case, positive φ in italic, disulfide-bonded cysteines are indicated with a cedilla, hydrogen bonds to other sidechains are indicated with a tilde, hydrogen bonds to mainchain amide bonds shown in bold and hydrogen bonds to mainchain carbonyls are underscored. Residues predicted to interact with FGFR2c based on the FGF8b-FGFR2c structure are underscored in blue. FGF8b residues that interact, but are not resolved in the FGF18 crystal structure are underscored in purple.

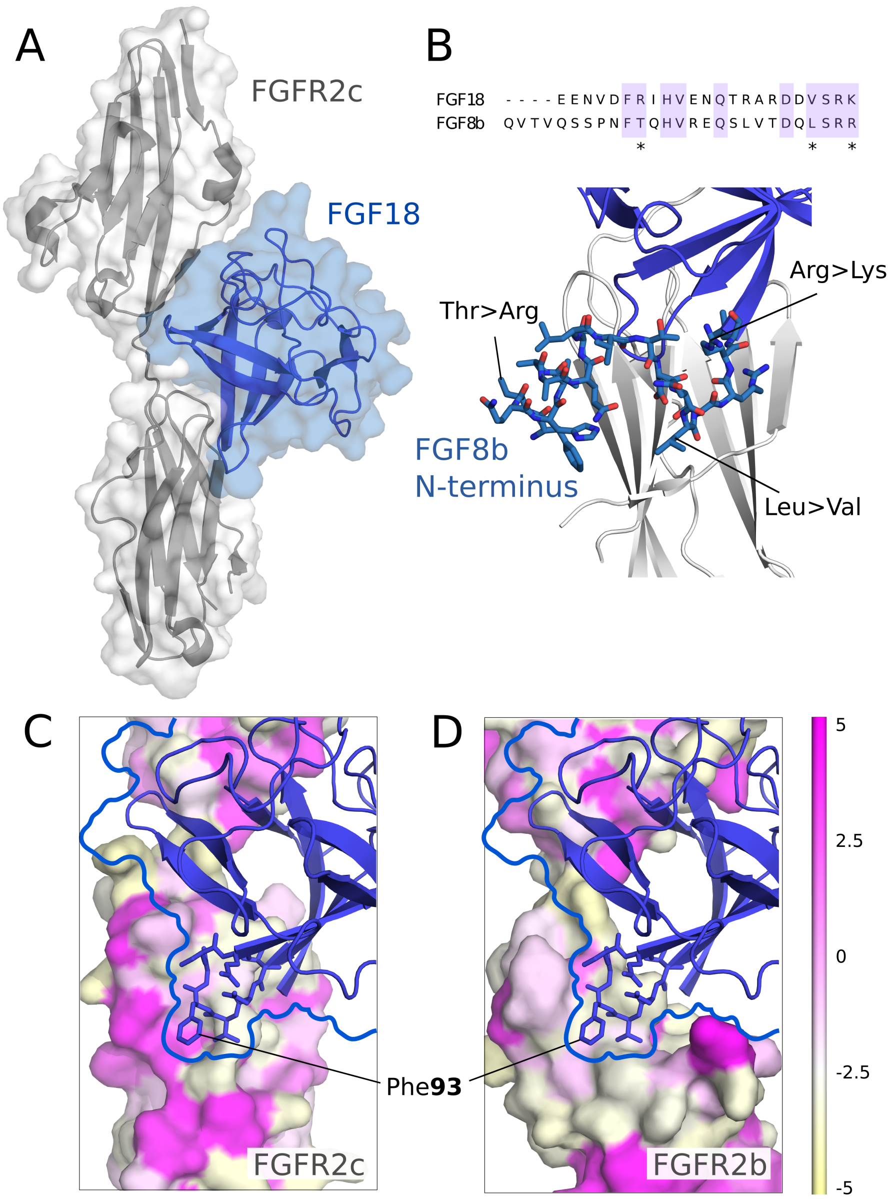

Fig. S4: Modelling of the FGF18-FGFR2 interaction. (A) The FGF18-FGFR2c complex modelled by superposition of FGF18 onto FGF8b (PDB ID: 2FDB). (B) The N-terminus of FGF18 is similar in sequence to FGF8b with three mutations in residues that engage with the receptor. (C) FGFR2c colored by a hydrophobicity scale (left) (Kyte and Doolittle, 1982). The hydrophobic β4-β5 loop of FGF18 (shown in stick representation) engages with the hydrophobic groove of the FGFR c-isoforms. (D) In FGFR b-isoforms, the β4-β5 loop of FGF18 would engage a more hydrophilic

region, explaining the preference of FGF18 for c-isoforms. In all panels FGF18 is shown in blue cartoon representation.

References

Chen VB, Arendall WB III, Headd JJ, Keedy DA, Immormino RM, Kapral GJ, Murray LW, Richardson JS, Richardson DC (2009) MolProbity: all-atom structure validation for macromolecular crystallography. Acta Crystallogr D Biol Crystallogr 66:12–21.

DeLano WL (2002) The PyMOL molecular graphics system.

Emsley P, Cowtan K (2004) Coot: model-building tools for molecular graphics. Acta Crystallogr D Biol Crystallogr 60:2126–2132.

Forbes SA, Bindal N, Bamford S, Cole C, Kok CY, Beare D, Jia M, Shepherd R, Leung K, Menzies A, Teague JW, Campbell PJ, Stratton MR, Futreal PA (2011) COSMIC: mining complete cancer genomes in the Catalogue of Somatic Mutations in Cancer. Nucleic Acids Res 39:D945–D950.

Heinig M, Frishman D (2004) STRIDE: a web server for secondary structure assignment from known atomic coordinates of proteins. Nucleic Acids Res 32:W500–W502.

Joosten RP, Joosten K, Cohen SX, Vriend G, Perrakis A (2011) Automatic rebuilding and optimization of crystallographic structures in the Protein Data Bank. Bioinformatics 27:3392–3398.

Krissinel E, Henrick K (2007) Inference of macromolecular assemblies from crystalline state. Journal of Molecular Biology 372:774–797.

Kyte J, Doolittle RF (1982) A simple method for displaying the hydropathic character of a protein. Journal of Molecular Biology.

Lise S, Buchan D, Pontil M, Jones DT (2011) Predictions of hot spot residues at protein-protein interfaces using support vector machines. PLoS ONE 6:e16774.

Maiti R, Van Domselaar GH, Zhang H, Wishart DS (2004) SuperPose: a simple server for sophisticated structural superposition. Nucleic Acids Res 32:W590–W594.

McCoy AJ, Grosse-Kunstleve RW, Adams PD, Winn MD, Storoni LC, Read RJ (2007) Phaser crystallographic software. J Appl Crystallogr 40:658–674.

Mizuguchi K, Deane CM, Blundell TL, Johnson MS, Overington JP (1998) JOY: protein sequence-structure representation and analysis. Bioinformatics 14:617–623.

Murshudov GN, Skubák P, Lebedev AA, Pannu NS, Steiner RA, Nicholls RA, Winn MD, Long F, Vagin AA (2011) REFMAC5 for the refinement of macromolecular crystal structures. Acta Crystallogr D Biol Crystallogr 67:355–367.

Ostrovsky O, Berman B, Gallagher JT, Mulloy B, Fernig DG, Delehedde M, Ron D (2002) Differential effects of heparin saccharides on the formation of specific

fibroblast growth factor (FGF) and FGF receptor complexes. J Biol Chem 277:2444–2453.

Pei J, Tang M, Grishin NV (2008) PROMALS3D web server for accurate multiple protein sequence and structure alignments. Nucleic Acids Res 36:W30–W34.

Peränen J, Rikkonen M, Hyvönen M, Kääriäinen L (1996) T7 vectors with modified T7lac promoter for expression of proteins in Escherichia coli. Anal Biochem 236:371–373.

Rose PW, Beran B, Bi C, Bluhm WF, Dimitropoulos D, Goodsell DS, Prlic A, Quesada M, Quinn GB, Westbrook JD, Young J, Yukich B, Zardecki C, Berman HM, Bourne PE (2011) The RCSB Protein Data Bank: redesigned web site and web services. Nucleic Acids Research 39:D392–D401.

Sherry ST, Ward MH, Kholodov M, Baker J, Phan L, Smigielski EM, Sirotkin K (2001) dbSNP: the NCBI database of genetic variation. Nucleic Acids Res 29:308–311.

Unni S, Huang Y, Hanson RM, Tobias M, Krishnan S, Li WW, Nielsen JE, Baker NA (2011) Web servers and services for electrostatics calculations with APBS and PDB2PQR. J Comput Chem 32:1488–1491.
